# Agonistic Anti-PDGF Receptor Autoantibodies from Patients with Systemic Sclerosis Impact Human Pulmonary Artery Smooth Muscle Cells Function *In Vitro*

**DOI:** 10.3389/fimmu.2017.00075

**Published:** 2017-02-08

**Authors:** Silvia Svegliati, Donatella Amico, Tatiana Spadoni, Colomba Fischetti, Doreen Finke, Gianluca Moroncini, Chiara Paolini, Cecilia Tonnini, Antonella Grieco, Marina Rovinelli, Ada Funaro, Armando Gabrielli

**Affiliations:** ^1^Clinica Medica, Dipartimento di Scienze Cliniche e Molecolari, Università Politecnica delle Marche, Ancona, Italy; ^2^Dipartimento di Scienze Mediche, Università di Torino, Torino, Italy

**Keywords:** systemic sclerosis, autoantibodies, vascular smooth muscle cells, platelet-derived growth factor, synthetic phenotype

## Abstract

One of the earliest events in the pathogenesis of systemic sclerosis (SSc) is microvasculature damage with intimal hyperplasia and accumulation of cells expressing PDGF receptor. Stimulatory autoantibodies targeting PDGF receptor have been detected in SSc patients and demonstrated to induce fibrosis *in vivo* and convert *in vitro* normal fibroblasts into SSc-like cells. Since there is no evidence of the role of anti-PDGF receptor autoantibodies in the pathogenesis of SSc vascular lesions, we investigated the biologic effect of agonistic anti-PDGF receptor autoantibodies from SSc patients on human pulmonary artery smooth muscle cells and the signaling pathways involved. The synthetic (proliferation, migration, and type I collagen gene α1 chain expression) and contractile (smooth muscle-myosin heavy chain and smooth muscle-calponin expression) profiles of human pulmonary artery smooth muscle cells were assessed *in vitro* after incubation with SSc anti-PDGF receptors stimulatory autoantibodies. The role of reactive oxygen species, NOX isoforms, and mammalian target of rapamycin (mTOR) was investigated. Human pulmonary artery smooth muscle cells acquired a synthetic phenotype characterized by higher growth rate, migratory activity, gene expression of type I collagen α1 chain, and less expression of markers characteristic of the contractile phenotype such as smooth muscle-myosin heavy chain and smooth muscle-calponin when stimulated with PDGF and autoantibodies against PDGF receptor, but not with normal IgG. This phenotypic profile is mediated by increased generation of reactive oxygen species and expression of NOX4 and mTORC1. Our data indicate that agonistic anti-PDGF receptor autoantibodies may contribute to the pathogenesis of SSc intimal hyperplasia.

## Introduction

Systemic sclerosis (SSc; scleroderma) is a multiorgan disorder characterized by microvasculature damage, circulating autoantibodies, and fibroblast activation leading to fibrosis of the skin and visceral organs (1–3).

Vascular involvement is an early and very likely primary event in the pathogenesis of scleroderma, precedes fibrosis, and is characterized by endothelial cell (EC) injury and dysfunction, altered capillary permeability, increased expression of adhesion molecules, abnormal secretion of vasoactive mediators, and activation of platelets and fibrinolytic pathways (4–7). Microvascular injury and damage lead to vascular remodeling with striking intimal hyperplasia (also called neointima) due to increase in cell number and extracellular matrix and marked luminal narrowing (7, 8). The process culminates in rarefaction of blood vessels on nailfold capillaroscopy of SSc patients with late-stage disease (9). Loss of microvasculature is associated with tissue hypoxia, which induces strong expression of VEGF and its receptors (10–12).

The source of the intimal cells in scleroderma vasculopathy is a matter of debate. Medial smooth muscle cells, pericytes, circulating fibrocytes, ECs, adventitial fibroblasts, and adventitial stem cells have all been held responsible (13). Furthermore, the exact cause of vascular injury in SSc is unknown, and it may include reactive oxygen species (ROS)-mediated damage, viral agents, anti-EC antibodies, ischemia–reperfusion events, cytotoxic T-lymphocytes, and antibody-dependent cellular cytotoxicity [see for review Ref. (14)]. A further mechanisms has been reported by Riemekasten et al. who detected serum autoantibodies against angiotensin II type I receptor and endothelin-1 type 1 receptor, which induced extracellular signal-regulated kinase 1/2 (ERK 1/2) phosphorylation and increased TGF-β gene expression in ECs and were associated with severe disease manifestations (15).

While over the years much attention has been devoted to EC injury in scleroderma, oddly enough no *in vitro* studies focused on smooth muscle cells that are rich in PDGF receptors (PDGFR) (16), a key signaling molecule in the pathogenesis of SSc fibrosis. High levels of PDGF and PDGF receptor β (PDGFR β) have been found in skin lesions from patients with scleroderma (17, 18) and may contribute to the differentiation of perivascular pericytes into vascular smooth muscle cells, fibroblasts, and myofibroblasts (19). The beneficial effects of selective inhibitors of PDGF signaling on dermal fibrosis (20, 21) and lung fibrosis (22) further indicate the importance of PDGF in scleroderma. Finally, the relevance of PDGFR has been further emphasized by the high prevalence of anti-PDGFRα autoantibodies in SSc sera (23, 24).

Anti-PDGFRα autoantibodies play a role in the pathogenesis of scleroderma since they convert *in vitro* normal fibroblasts into SSc-like cells *via* the ROS, RAS, and ERK 1/2 pathway (23–26) and are capable to induce fibrosis *in vivo* (27). No report has, however, described their effect on human smooth muscle cells, and since a better understanding of the molecular mechanisms involved in scleroderma vascular events could help to prevent severe complications such as digital ulcers, pulmonary hypertension, and renal crisis, which are responsible for a substantially reduced survival and impaired quality of life (28–30), we decided to investigate the biological effects of SSc agonistic anti-PDGFR autoantibodies on human pulmonary artery smooth muscle cells (HPASMC) *in vitro*.

## Materials and Methods

### Patients and Biological Samples

Serum samples were obtained from 11 SSc patients (2 were male and 9 were female) with median age of 56 years (range, 43–73 years), and median disease duration (defined as the time from the onset of the first non-Raynaud’s phenomenon clinical manifestation) was 7 years (2–21). Diagnosis was made following the ACR/EULAR preliminary criteria for the classification of SSc (31). Five patients had limited cutaneous SSc and six diffuse cutaneous SSc according to LeRoy et al. (32). Six patients were antitopoisomerase I positive; two patients were anticentromere positive, and three were ANA positive but lacked specific SSc autoantibodies. In the whole group, median PAPs detected by echocardiography was 30 mmHg (range, 28.5–36.5 mmHg) (Table 1). The study was approved by the local ethic committee. A written informed consent was obtained from all patients. At the time of the investigation, patients who had never been on immunosuppressive therapy had not received any treatment for the previous 6 weeks. Control sera were obtained from 10 age-, sex-, and race-matched normal, non-smoking, healthy volunteers. All subjects provided informed consent and the study protocol was approved by the Institutional Ethics Committee of Università Politecnica delle Marche.

**Table 1:** **Clinical characteristics of systemic sclerosis patients (*n* = 11)**.

Male/female	2/9
Mean age, years (range)	56 (43–73)
Mean modified Rodnan skin score (range)	11 (4–30)
Subset ISSc/dSSc	5/6
Median disease duration, years (range)	7 (2–21)
Antitopoisomerase I-positive patients	6
Anticentromere-positive patients	2
ANA-positive patients	3

### Antibody Purification

Immunoglobulins were purified from serum using gravity flow column packed with protein A/G agarose following manufacturer instruction (Pierce). Concentrations of all samples were calculated based on absorbance at 280 nm. The eluted IgG fractions that contain the highest absorbance were subjected to buffer exchange with PBS using desalting column, a size-exclusion chromatography with an average molecular weight exclusion limit of 5 KDa (Pierce). Purity of IgG preparations was checked by Coomassie blue staining, and PDGF and TGF-β contaminations were ruled out by immunoblotting with biotinylated anti-human PDGF-BB and anti-human TGF-β antibodies (Abcam), respectively, with detection limit of 0.1 ng cytokine/200 μg IgG.

### Human Monoclonal Anti-PDGFRα Autoantibodies

Agonistic (V_H_PAM-V_κ_16F4) and non-agonistic (V_H_PAM-V_κ_13B8) human monoclonal autoantibodies targeting PDGFRα were generated from SSc memory B cells as already described (24).

### Cell Cultures

Human pulmonary artery smooth muscle cells (HPASMC) were purchased from Lonza and maintained in Medium 231 supplemented with smooth muscle growth medium (SMGM) and 100 U/ml penicillin/streptomycin (all from Life Technologies). Cells between passages 3 and 9 were used in all experiments performed after 24 h of starvation (0.1% SMGM).

Total IgGs were isolated from each patient and control and were singly tested in triplicate. The same conditions were applied to human monoclonal antibodies. Each experiment was performed at least three times. Means of all experiments are shown.

### ROS Detection

Relative changes in intracellular ROS were monitored using the fluorescent probe 2′, 7′-dichlorfluorescein-diacetate (DCFH-DA; Life Technologies). Cells were plated at 2.5 × 10^5^/ml and then incubated with scleroderma (SSc IgG) or normal subjects IgG (N IgG) (200 µg/ml), PDGF (15 ng/ml), or human monoclonal anti-PDGFRα autoantibodies (10 µg/ml) for 15 min and then stained with 10 µM DCFH-DA (Life Technologies) for 10 min at 37°C. Fluorescence was read using a plate reader fluorimeter (Victor 2, Perkin Elmer).

For confocal microscopy experiments, cells were seeded on coverslides and treated as described earlier. Cells were loaded with 10 µM DCFH-DA for 30 min, washed with PBS, and then visualized on a confocal microscope Eclipse C1 (Nikon). Data were analyzed by ImageJ software.

The intracellular content of ROS was also measured at a single-cell level by flow cytometry (FACS, Becton Dickinson). Briefly, cells were detached by trypsin, washed with PBS, and incubated with 5 µM DCFH-DA for 20 min at 37°C. Cells were then washed and resuspended in 1 ml PBS. For each sample, 10,000 events were collected, and intracellular ROS formation was detected as a result of oxidation of DCFH-DA at a wavelength of 520 nm. Data analysis was carried out using the WinMDI software.

Where indicated, the inhibitor of flavoprotein-dependent oxidases diphenyleneiodonium (DPI; 10 µM, Calbiochem), a general ROS inhibitor *N*-acetyl-cysteine (NAC; 10 mM, Sigma), and the PDGFR chemical inhibitor AG1296 (2 µM, Calbiochem) were added 1 h before stimulation.

### Proliferation Assay

Cell proliferation was determined by 5-bromo-2′-deoxyuridine (BrdU) incorporation assay (Roche). Briefly, HPASMC were seeded in 96-well pates at a density of 2.5 × 10^5^ cells/ml and then treated with SSc IgG or N IgG (200 µg/ml), PDGF (15 ng/ml), or human monoclonal anti-PDGFRα autoantibodies (10 µg/ml) for 48 h. Cells were pulsed for 6 h with BrdU, and plates were analyzed in a plate reader at 450 nm (Victor 2, PerkinElmer).

### Migration Assay

Migration was studied by the wound scratch assay (33). Briefly, the cell monolayer was scraped by a straight line to create a “scratch” with a pipette tip. Cells were incubated for 24 h with SSc IgG, N IgG (200 µg/ml), PDGF (15 ng/ml), or human monoclonal anti-PDGFRα autoantibodies (10 µg/ml) in the presence or absence of inhibitors. Mitomycin was added to inhibit cell proliferation. Unstimulated cells were used as negative controls. The percentage of cells repopulating the scratch wound was assessed after 24 h.

### Reverse Transcriptase Polymerase Chain Reaction

Total RNA was isolated from HPASMC with Pure Link RNA Minikit (Life Technologies) and reverse transcribed using IScript cDNA Synthesis Kit (Bio-Rad Laboratories) according to the instructions of the manufacturer. Gene expression was quantified by SYBR Green real-time PCR in a iCycler iQ™ real-time PCR Detection System (Bio-Rad). Specific primer pairs for each gene were designed with the Universal ProbeLibrary Assay Design Centre by Roche Applied Science and were as follows: NOX1 5′-CTGTTTGTGGATGCCTTCCT-3′ (forward), 5′-TGTGGAAGGTGAGG-TTGTGA-3′ (reverse); NOX2 5′-TCACTTCCTCCACCAAAACC-3′ (forward), 5′GGGATTGGGCATTCCTTTAT-3′ (reverse); NOX4 5′-CTTCCGTTGGTTTGCAGATT-3′ (forward), 5′-GAATTGGGCCACAACAGA-3′ (reverse); type I collagen α1 chain (COL1A1) 5′-AGGGCCAAGACGAAGACATC-3′ (forward), 5′-AGATCACGTCATCGCACAACA-3′ (reverse); smooth muscle-myosin heavy chain (SM-MHC) 5′-CAGGCGTTCCGCCAACGCTA-3′ (forward), 5′-TCCCGTCCATGAAGCCTTTGG-3′ (reverse); smooth muscle-calponin (sm-calponin) 5′-TTTTGAGGCCAACGACCTGT-3′ (forward), 5′-TCCTTTCGTCTTCGCCATG-3′ (reverse); and GAPDH 5′-TGCACCACCAACTGCTTAGC-3′ (forward), 5′-TGGGATTTCCATTGATGA-CAAGC-3′ (reverse).

GAPDH was used to test the quality of cDNA and as a house keeping gene in real-time PCR. The threshold cycle (Ct) was used to detect the increase in the signal associated with an exponential growth of PCR product during the log-linear phase. The relative expression was calculated using the following formula: 2^−ΔΔ^Ct. The ΔCt validation experiments showed similar amplification efficiency for all templates used (difference between linear slopes for all templates less than 0.1).

### Immunoblotting

Cells were lysed in RIPA buffer [1× PBS, 1% non-idet P-40, 0.5% sodium deoxycholate, 0.1% sodium dodecyl sulfate (SDS), 2 mM sodium orthovanadate] supplemented with a cocktail of protease inhibitors (Sigma). Proteins were separated on 4–12% gradient SDS–polyacrylamide gels, transferred onto nitrocellulose membranes, and immunoblotted with antibodies against NOX4, β-actin (Santa Cruz Biotechnology), and phospho mTOR and mTOR (Cell Signalling). Signals were detected by chemiluminescence (Pierce). Densitometric analysis was performed with Quantity One software (BioRad). Where indicated rapamycin (10 nM) was added 48 h before stimulation.

### NOX4 Intracellular Expression

For intracellular staining assay, HPASMC were treated with SSc IgG or N IgG (200 µg/ml) and PDGF (15 ng/ml) for 24 h and then permeabilized with Cyto Fix/Perm (Becton Dickinson). Cells were detached by trypsin, washed with PBS, and incubated with antibody against NOX4 (AbCam) for 24 h at 37°C. Cells were then washed and incubated with FITC-conjugated secondary antibody and resuspended in 1 ml PBS. For each sample, 10,000 events were collected. Data analysis was carried out using the WinMDI software.

### Small Interfering RNA (siRNA) Experiments

Nox4 was silenced using siRNA oligoribonucleotides (Qiagen) using Lipofectamine™ 2000 reagent (Life Technologies) following the manufacturer’s instructions. Briefly, 10 µM NOX4 siRNA or scrambled oligos and Lipofectamine 2000 were mixed in serum-free Medium 231 without antibiotics and then added to the cells. Medium was changed after 4 h. Gene silencing and ROS production were monitored 72 h after transfection.

### Statistical Analysis

Statistical analysis was performed using GraphPad Prism version 4.0 by two-tailed Student’s *t*-test. *P* values less than 0.05 were considered significant.

## Results

### Agonistic Anti-PDGFRα Receptor Autoantibodies from SSc Patients Induce Increased ROS Generation in HPASMC

**Figure 1:**
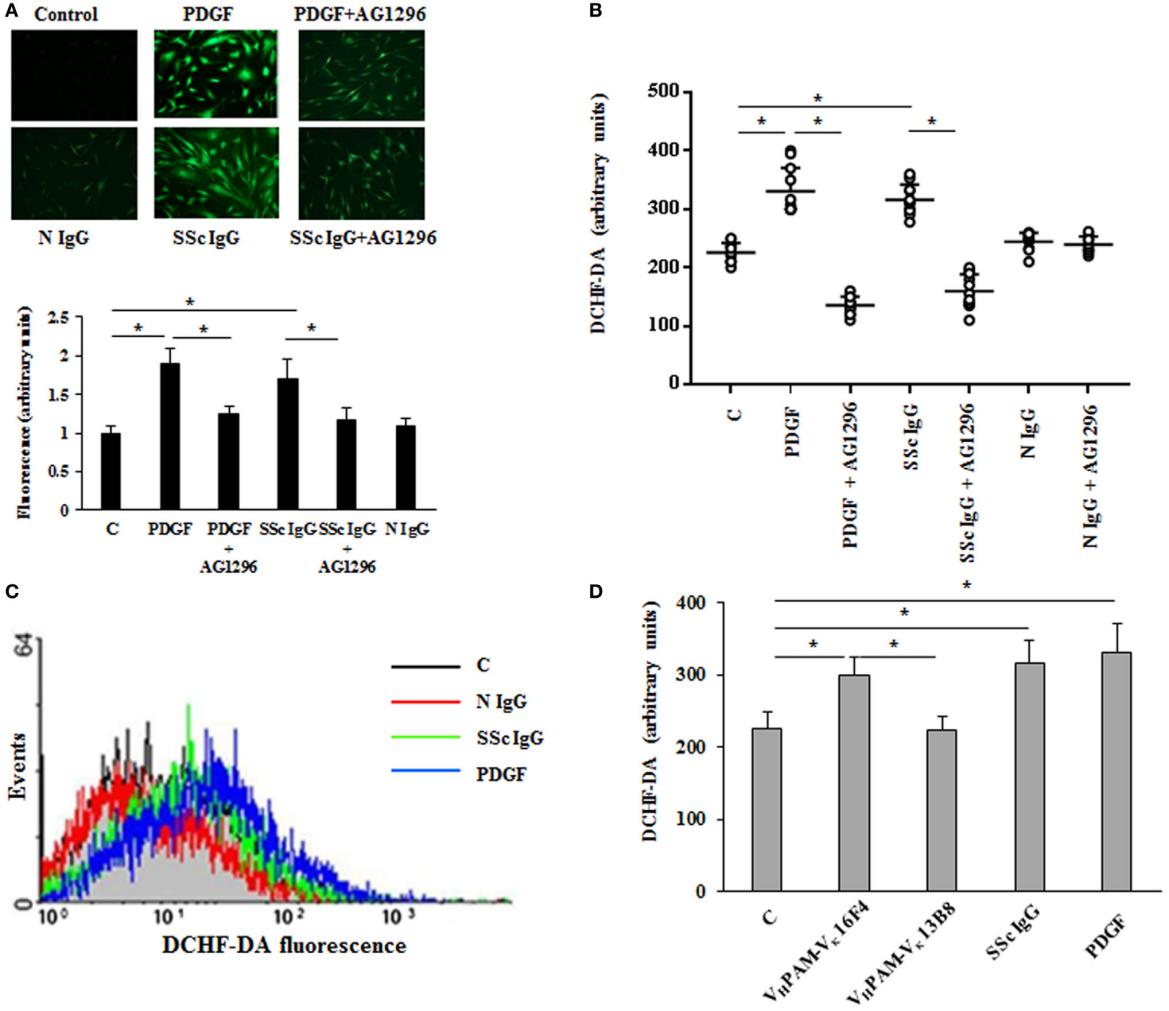
**Agonistic anti-PDGF receptor autoantibodies from systemic sclerosis (SSc) patients induce increased reactive oxygen species (ROS) generation in HPASMC**. HPASMC were exposed to PDGF (15 ng/ml) or IgG isolated from each SSc patients (SSc IgG; *n* = 11; 200 µg/ml) or normal controls (N IgG; *n* = 10; 200 µg/ml) for 15 min in the presence or absence of AG1296 (2 µM) that was added 1 h before stimulation with Ig. Unstimulated cells were used as a control (C). **(A)** Generation of intracellular ROS was evaluated by fluorescence microscopy using DCHF-DA as a oxidative probe. Representative images of three independent experiments are shown (upper panel). Scale bar = 100 µm. The fluorescence was quantified by ImageJ software (lower panel). The IgG from each subject was tested. Data are presented as mean ± SD of three independent experiments. *p < 0.05. **(B)** Intracellular ROS production measured by plate reader fluorimeter. The IgG from each subject was tested in triplicate. Data are presented as mean ± SD of three independent experiments. *p < 0.05. **(C)** ROS production of untreated cells (C) (black line), SSc IgG-treated cells (green line), N IgG-treated cells (red line), and PDGF-treated cells (blue line) by FACS analysis. A representative histogram of three independent experiments is shown. **(D)** HPASMC were stimulated for 15 min with PDGF (15 ng/ml), SSc IgG (200 µg/ml), and the agonistic (V_H_PAM-V_κ_16F4) and non-agonistic (V_H_PAM-V_κ_13B8) human monoclonal anti-PDGFRα autoantibody (10 µg/ml each). Unstimulated cells were used as a control (C). ROS generation was evaluated by fluorometry. Data are presented as mean ± SD of three independent experiments. *p < 0.05.

Since the pathogenesis of scleroderma is characterized by an abnormal generation of ROS [for review, see Ref. (34)] and several lines of evidence implicate oxidative stress in the pathogenesis of PAH (35), we exploited our previous demonstration that agonistic anti-PDGFRα autoantibodies isolated from SSc sera induce an abnormal generation of ROS in normal fibroblasts *via* NOX (23, 24, 36). Hence, HPASMC were stimulated *in vitro* with IgG isolated from serum of distinct scleroderma patients (SSc IgG; *n* = 11) or distinct normal controls (N IgG; *n* = 10) (200 µg/ml, 15 min) and then incubated with the peroxide-sensitive fluorophore DCHF-DA. Confocal microscopy (Figure 1A), plate reader fluorimeter (Figure 1B), or FACS analysis (Figure 1C) showed that the levels of ROS, induced by PDGF and SSc IgG, were higher than the levels obtained in unstimulated cells (*p* < 0.05) and were inhibited by preincubating cells with PDGFR tyrosine kinase inhibitor AG 1296 (2 µM, 1 h) (*p* < 0.05), indicating that ROS production occurred through PDGFRα. The amount of ROS generated by N IgG was not significantly different from that obtained in unstimulated cells (Figures 1A–C).

Systemic sclerosis IgGs contain a multitude of SSc disease-specific and non-specific antibodies, including agonistic autoantibodies against angiotensin II receptor type 1 and the endothelin receptor type A (37). Thus, to demonstrate that the findings reported above are related to stimulatory antibodies targeting PDGFRα, HPASMC were exposed to combinatorial human monoclonal anti-PDGFRα autoantibody V_H_PAM-V_κ_16F4 (10 µg/ml), which binds and stimulate the PDGFRα *in vitro*, and to antibody V_H_PAM-V_κ_13B8 (10 µg/ml), which binds but does not stimulate the receptor *in vitro* (24). Figure 1D shows that the agonistic antibody V_H_PAM-V_K_16F4-stimulated cells produced significantly larger amount of ROS compared to unstimulated cells and V_H_PAM-V*_K_*13B8-treated HPASMC. No difference was observed between V_H_PAM-V_K_13B8-treated and unstimulated cells.

### Enhanced Migration and Proliferation of HPASMC by SSc IgG through PDGFR

**Figure 2:**
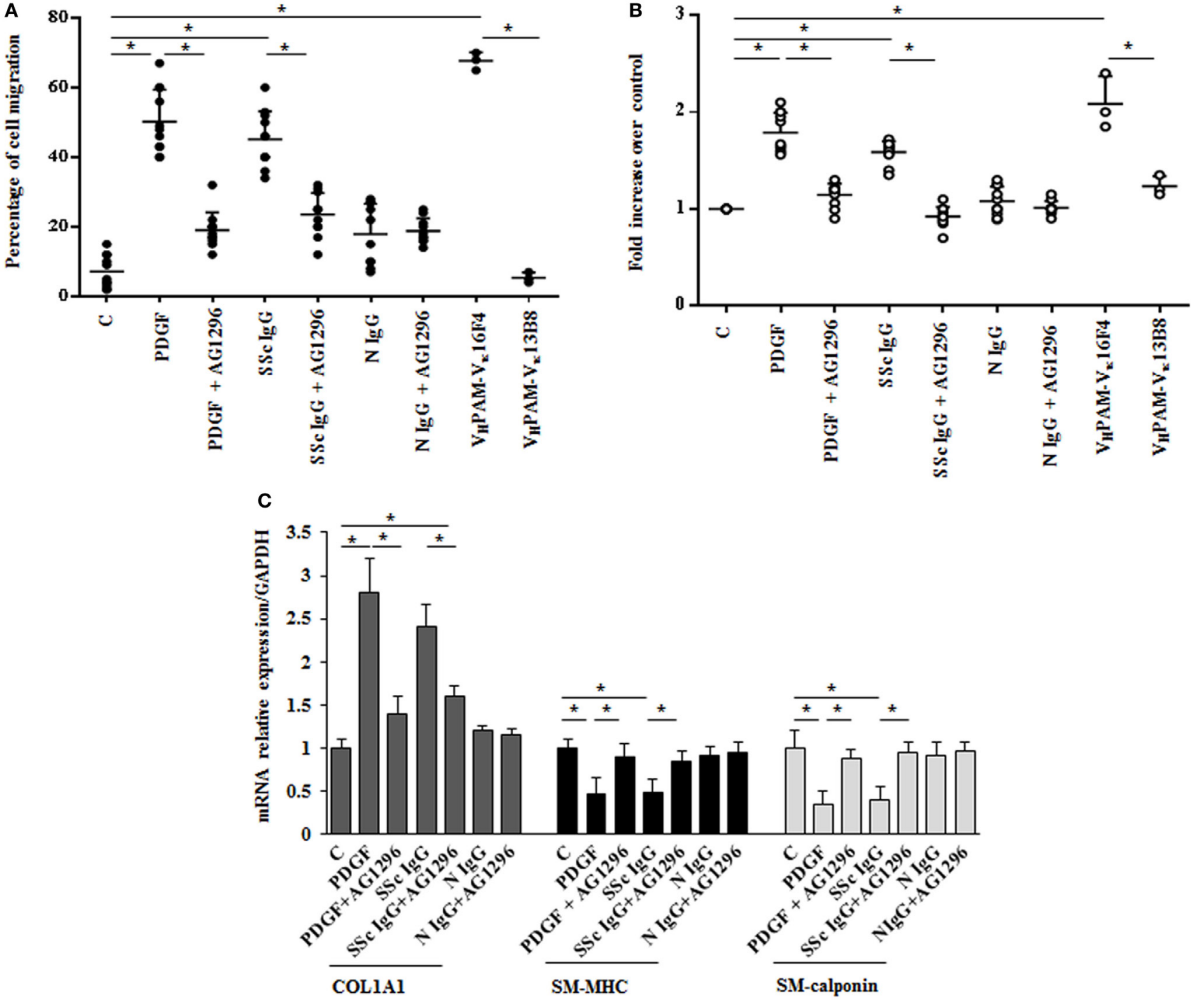
**Biological effects induced by systemic sclerosis (SSc) IgGs are mediated by PDGF receptor α (PDGFRα)**. **(A)** Migration was analyzed by the wound healing scratch test. Cells were scraped and stimulated with PDGF (15 ng/ml), distinct SSc IgG (200 µg/ml, *n* = 11), or distinct N IgG (200 µg/ml, *n* = 10) in the presence or absence of AG1296 or with agonistic (V_H_PAM-V_κ_16F4) and non-agonistic (V_H_PAM-V_κ_13B8) human monoclonal anti-PDGFRα autoantibody. After 24 h, the gap size was measured and expressed as a percentage of the original wound. The IgG from each subject was tested in triplicate. Data are presented as mean ± SD of three independent experiments. *p < 0.05. **(B)** HPASMC treated with PDGF (15 ng/ml), single-tested SSc IgG (200 µg/ml; *n* = 11), or N IgG (200 µg/ml; *n* = 10) or with the human monoclonal anti-PDGFRα autoantibody V_H_PAM-V_k_16F4 and V_H_PAM-V_κ_13B8 for 48 h in the presence or the absence of AG1296 (2 µM). Proliferation was determined by 5-bromo-2′-deoxyuridine assay and expressed as fold increase over controls. The IgG from each subject were tested in triplicate. Data are presented as mean ± SD of five independent experiments. *p < 0.05. **(C)** HPASMC were treated with AG1296 (2 µM) for 1 h and then exposed to PDGF (15 ng/ml), SSc IgG (200 µg/ml; *n* = 11), or N IgG (200 µg/ml; *n* = 10) for 24 h. COL1A1, smooth muscle-myosin heavy chain, and SM-calponin mRNA levels were measured by real-time PCR. Data are presented as mean ± SD of three independent experiments. *p < 0.05. The IgG from each subject was single tested.

The *in vitro* scratch assay was used to study the effect of SSc IgG on HPASMC migration (33). Incubation with PDGF (15 ng/ml) or SSc IgG (200 µg/ml) for 24 h enhanced migratory ability of HPASMC compared to cells not stimulated used as controls (50 and 45%, respectively, over control cells, *p* < 0.05) (Figure 2A). Migration after N IgG was not statistically different from that of unstimulated HPSMC. The addition of AG1296 (2 µM, 1 h) significantly reduced PDGF- or SSc IgG-induced migration (*p* < 0.05). AG1296-treated cells did not differ from unstimulated or N IgG-treated cells (*p* = n.s.) (Figure 2A).

Agonistic V_H_PAM-V_K_16F4 (10 µg/ml) significantly stimulated HPSMC migration when compared to unstimulated cells and V_H_PAM-V*_K_*13B8-treated cells (60 and 62%, respectively; *p* < 0.05) (Figure 2A). No difference was detected between unstimulated and V_H_PAM-V_K_13B8-treated cells (*p* = n.s.) (Figure 2A).

HPASMC were incubated with PDGF (15 ng/ml) or IgG isolated from serum of distinct scleroderma patients (SSc IgG; *n* = 11) or normal controls (N IgG; *n* = 10) (200 µg/ml) for 48 h, and cell proliferation was determined by monitoring BrdU incorporation for 6 h. SSc IgG and PDGF significantly increased the number of BrdU-positive cells compared to unstimulated cells (1.8- and 1.58-fold, respectively; *p* < 0.05). This effect was reverted by incubating HPSMC with AG1296 (2 µM, 1 h) (Figure 2B). No difference was demonstrated between unstimulated cells and N IgG-treated cells. These findings were confirmed when cells were exposed to VH_PAM_-V*_K_*16F4 and proliferation compared to that obtained with VH_PAM_-V*_K_*13B8 (Figure 2B).

To determine whether SSc IgG contributed to establish a phenotypic modulation of HPASMC cells favoring a transition from contractile to synthetic state, we detected the expression of transcripts of COL1A1 and smooth muscle cell-specific contractile markers, SM-MHC, and SM-calponin mRNA (38). PDGF and SSc IgG increased COL1A1 gene expression (2.8 ± 0.7- and 2.4 ± 0.2-fold, respectively, when compared to unstimulated cells; *p* < 0.05) and decreased SM-MHC expression (0.46 ± 0.2- and 0.5 ± 0.1-fold, respectively, when compared to unstimulated cells; *p* < 0.05) and SM-calponin transcription (0.35 ± 016- and 0.4 ± 0.16-fold, respectively, when compared to unstimulated cells, respectively; *p* < 0.05) consistent with a synthetic phenotype (Figure 2C). Cells incubated with N IgG showed the same levels of transcripts of unstimulated cells. Experiments performed in the presence of AG1296 confirmed the role of PDGFR as a primary target of SSc IgG. All these findings are consistent with the induction of a synthetic phenotype in HPSMC by anti-PDGFRα autoantibodies.

**Figure 3:**
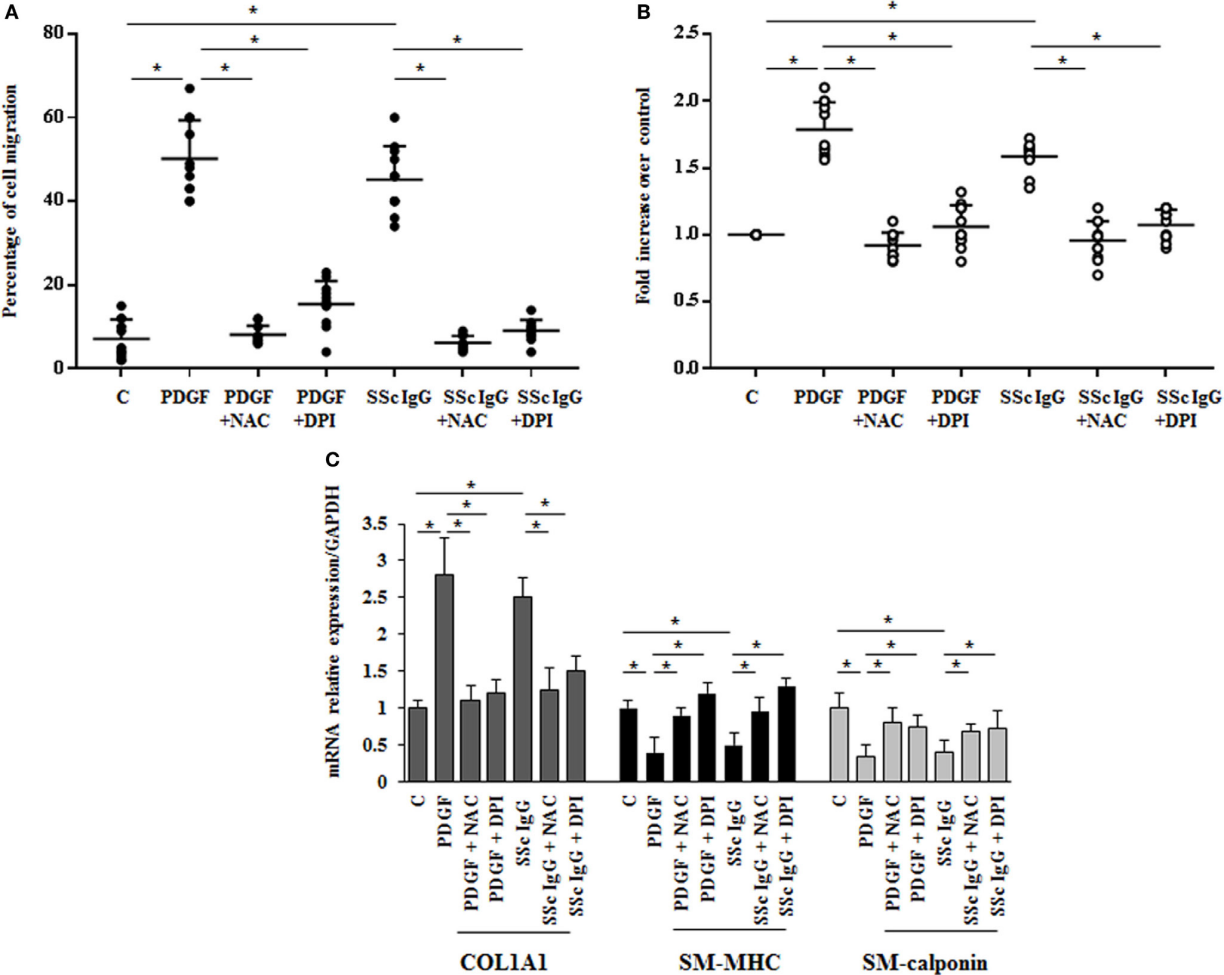
**Biological effects induced by systemic sclerosis (SSc) IgG are mediated by reactive oxygen species**. **(A)** HPASMC were scraped and stimulated with PDGF (15 ng/ml) or IgG isolated from each SSc patient (200 µg/ml; *n* = 11) and single tested in the presence or absence of *N*-acetyl-cysteine (NAC) (10 mM) or diphenyleneiodonium (DPI) (10 µM). After 24 h, the gap size was measured and expressed as a percentage of the original wound. Data are presented as mean ± SD of five independent experiments. *p < 0.05. **(B)** HPASMC treated with PDGF (15 ng/ml) and SSc IgG (200 µg/ml) for 48 h in the presence or absence of NAC (10 mM) or DPI (10 µM) for 1 h. Proliferation was determined by 5-bromo-2′-deoxyuridine assay and expressed as fold increase over controls. Data are presented as mean ± SD of five independent experiments. *p < 0.05. The IgG from each subject was single tested. **(C)** HPASMC were treated with NAC (10 mM) or DPI (10 µM) for 1 h and then exposed to PDGF (15 ng/ml) and distinct SSc IgG (200 µg/ml, *n* = 11) for 24 h. COL1A1, smooth muscle-myosin heavy chain, and SM-calponin mRNA levels were measured by real-time PCR. Data are presented as mean ± SD of three independent experiments. *p < 0.05.

The stimulation of HPASMC by single SSc IgG occurred through enhanced ROS generation, since the addition of the generic antioxidant NAC (10 mM) and the selective NADPH oxidase inhibitor DPI (10 µM) for 1 h before stimulation significantly prevented cell migration and proliferation induced by PDGF (15 ng/ml) and SSc IgG (200 µg/ml; *n* = 11) (*p* < 0.05) (Figures 3A,B). Moreover, the upregulation of COL1A1 and the reduction of mRNA transcript for SM-MHC and SM-calponin induced by PDGF and SSc IgG were reverted by the addition of NAC and DPI (Figure 3C).

These results indicate that SSc IgG induce phenotypic modulation and affect the rate of proliferation and migration of HPASMC through ROS produced by NOX activation.

### Enhanced Expression of NOX4 by SSc IgG in HPASMC

**Figure 4:**
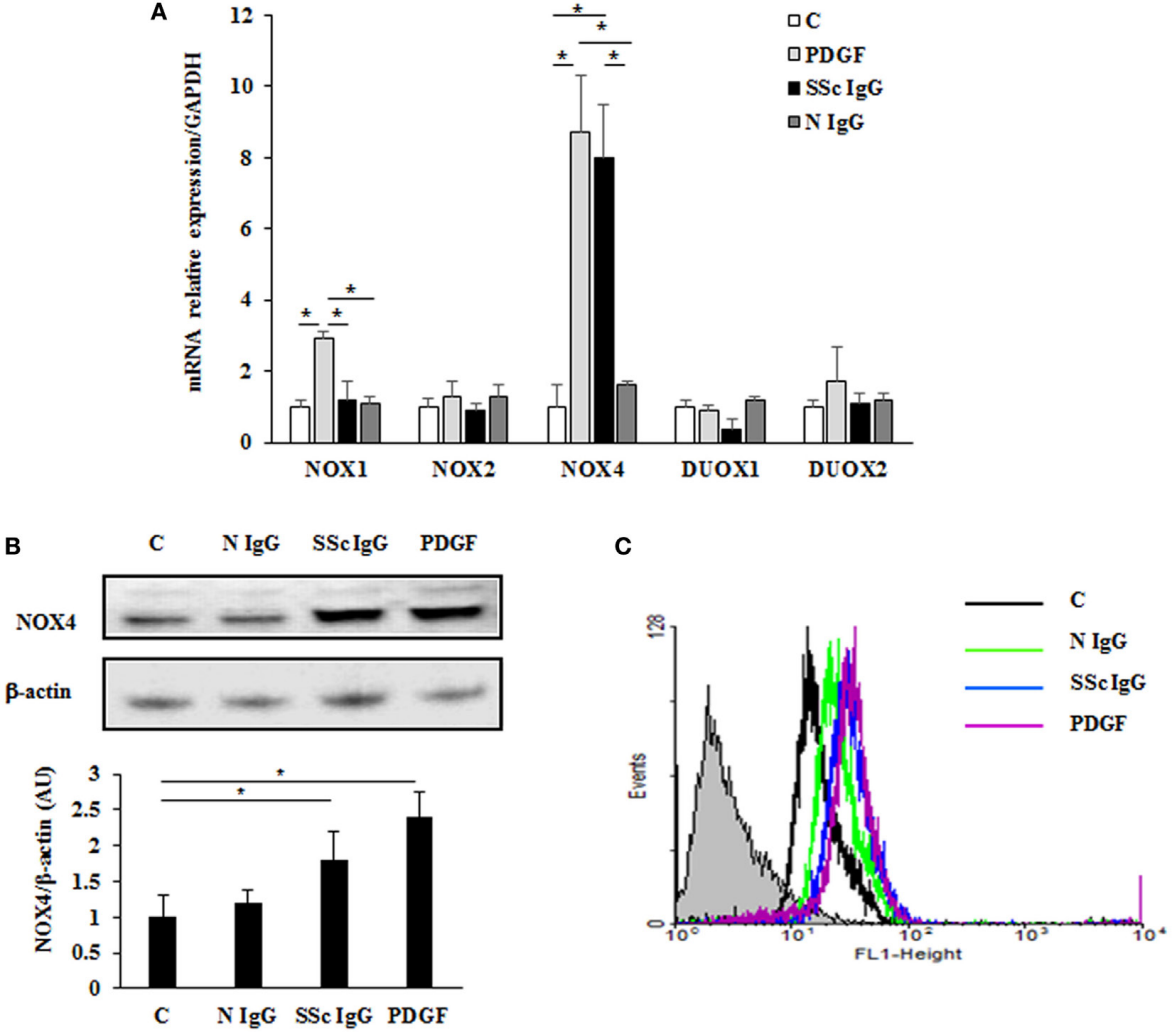
**Induction of NOX4 levels by systemic sclerosis (SSc) IgG**. **(A)** Real-time PCR analysis of NOX isoforms in HPASMC treated with PDGF (15 ng/ml) or IgG isolated from distinct SSc patients (200 µg/ml; *n* = 11) or distinct N IgG (200 µg/ml; *n* = 10) and single tested for 24 h. Data are presented as mean ± SD of three independent experiments. *p < 0.05 compared to controls. **(B)** Total cell lysates from HPASMC stimulated with PDGF (15 ng/ml), single SSc IgG (200 µg/ml; *n* = 5), or single N IgG (200 µg/ml; *n* = 5) for 24 h were analyzed by immunoblotting with a specific antibody against NOX4. β-actin was used as a control for normalization. One representative experiment is shown in the upper panel. Densitometric analysis from three independent experiments is reported in the lower panel. Data are presented as mean ± SD from three independent experiments. *p < 0.05. **(C)** One representative FACS analysis of NOX4 expression is shown. Cells were stimulated with PDGF (15 ng/ml), distinct SSc IgG (200 µg/ml; *n* = 11), or N IgG (200 µg/ml; *n* = 10); permealized; and then labeled with anti-NOX4 and secondary FITC-conjugated antibody.

To identify which NOX isoforms were upregulated in stimulated HPASMC, we treated HPASMC with PDGF (15 ng/ml) or single SSc IgG (200 µg/ml; *n* = 11) or IgG from normal subjects (N IgG; *n* = 10) (200 µg/ml) for 24 h, and the mRNA levels of the different NOX isoforms were assessed by real-time PCR. After stimulation, NOX4 mRNA levels were significantly upregulated compared to unstimulated cells (8.7 ± 1.5 and 8 ± 1.6 fold, respectively, *p* < 0.05), whereas NOX2, DUOX1, and DUOX2 were unaffected by the treatment (Figure 4A). PDGF, but not SSc IgG, increased NOX1 mRNA relative to control (2.9 ± 0.2-fold) but, as demonstrated elsewhere (36), the silencing of NOX1 did not influence the ROS generation in these cells (data not shown).

A significant increase of NOX4 protein expression compared to basal conditions was observed by immunoblotting (Figure 4B; *p* < 0.05) and FACS analysis (Figure 4C; *p* < 0.05) after a 24-hour exposure to PDGF or SSc IgG, whereas no effect was observed after N IgG addition (200 µg/ml; *n* = 10).

**Figure 5:**
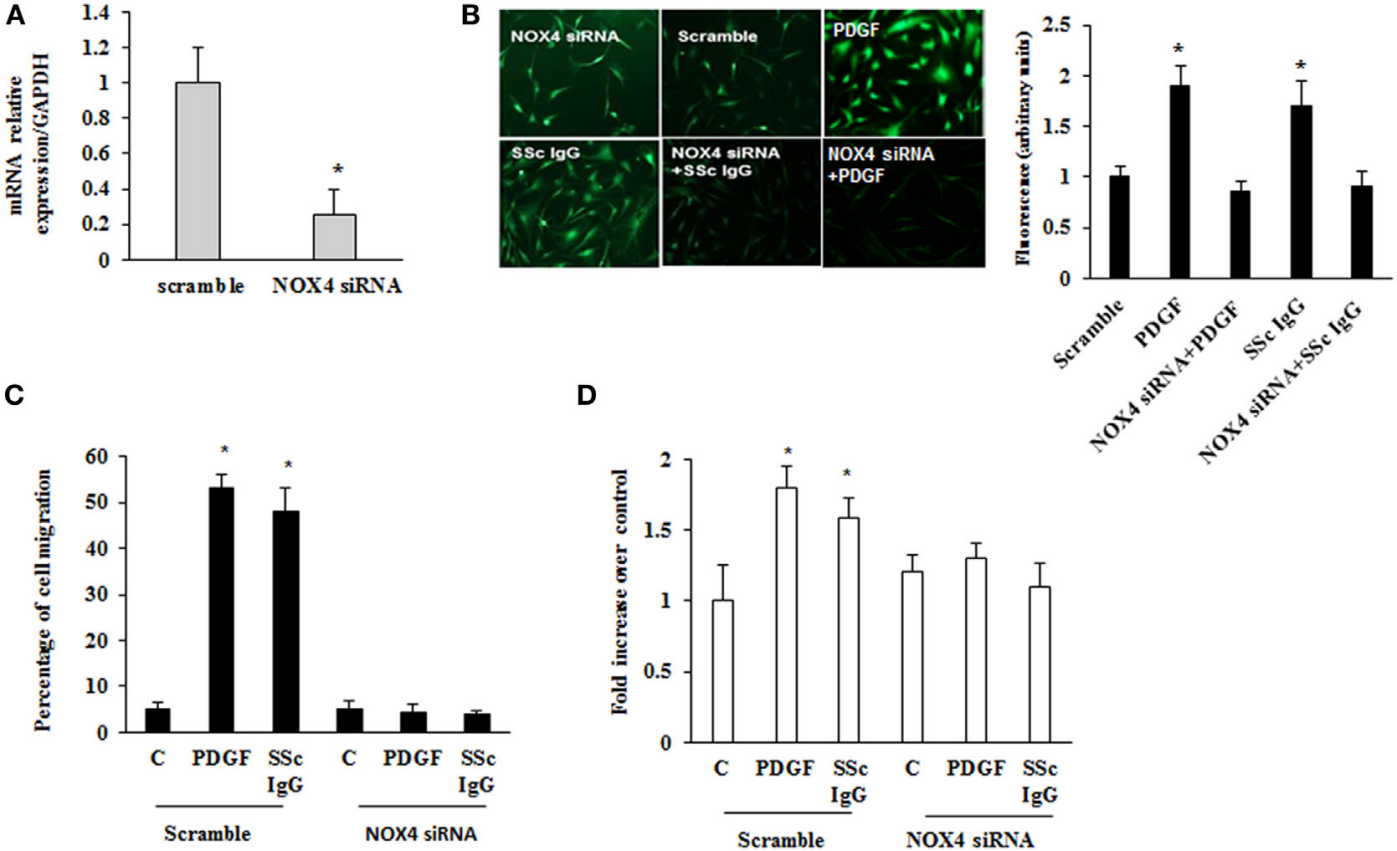
**Inhibition of systemic sclerosis (SSc) IgG biological effects by silencing NOX4**. **(A)** HPASMC were transfected with NOX4 or scramble siRNA for 24 h, and NOX4 mRNA levels were analyzed by real-time PCR analysis. Data are presented as mean ± SD of three independent experiments. *p < 0.05 compared to cells transfected with scramble siRNA. **(B)** HPASMC were transfected with NOX4 siRNA or scramble siRNA for 24 h and then were stimulated with PDGF (15 ng/ml) or distinct SSc IgG (200 µg/ml; *n* = 11) for 15 min. Cells were incubated with DCHF-DA, and free radical production was evaluated by fluorescence microscopy. Representative images of three independent experiments are shown. The fluorescence was quantified using ImageJ software (right panel). **(C)** HPASMC were transfected with NOX4 or scramble siRNA for 24 h, scraped, and stimulated with PDGF (15 ng/ml) or single SSc IgG (200 µg/ml). After 24 h, the gap size was measured and expressed as a percentage of the original wound. Data are presented as mean ± SD of five independent experiments. *p < 0.05. **(D)** Transfected cells with NOX4 or scramble siRNA for 24 h were treated with PDGF (15 ng/ml) or single SSc IgG (200 µg/ml; *n* = 11) for 48 h. Proliferation was determined by 5-bromo-2′-deoxyuridine assay and expressed as fold increase over controls. Data are presented as mean ± SD of five independent experiments. *p < 0.05 compared to unstimulated cells.

**Figure 6:**
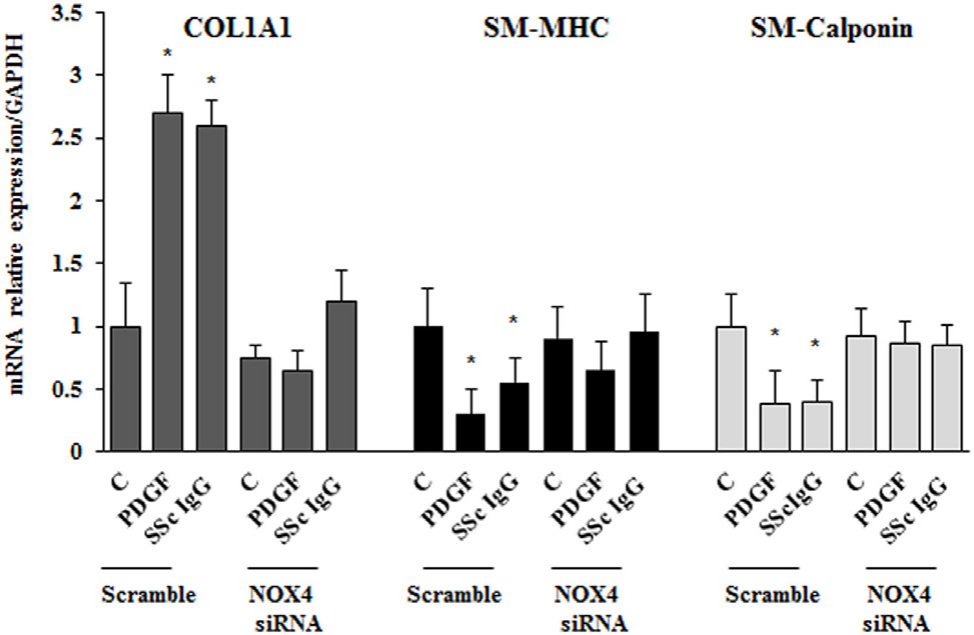
**NOX4 silencing drives a synthetic phenotype in HPASMC**. Transfected cells with NOX4 siRNA or scramble siRNA for 24 h were treated with PDGF (15 ng/ml) or systemic sclerosis IgG (200 µg/ml; *n* = 11) for 24 h. COL1A1, smooth muscle-myosin heavy chain, and SM-calponin mRNA levels were measured by real-time PCR. Data are presented as mean ± SD of three independent experiments. *p < 0.05 compared to unstimulated cells.

To dissect the role of NOX4 in ROS generation after PDGF or SSc IgG stimulation, we silenced NOX4 using small interfering RNA (siRNA) (*p* < 0.05) (Figure 5A). The specific siRNA selectively prevented ROS generation in HPASMC stimulated with either 15 ng/ml PDGF or 200 µg/ml SSc IgG for 15 min (Figure 5B) and reverted the effects of PDGF or SSc IgG on migration and proliferation (Figures 5C,D) compared to unstimulated cells. Silencing of NOX4 inhibited PDGF, SSc IgG induction of COL1A1, and downregulation of SM-MHC and SM-calponin, indicating a regulatory role of NOX4 in the phenotypic modulation of HPASMC (Figure 6).

### mTOR Modulated the Effects of SSc IgG

**Figure 7:**
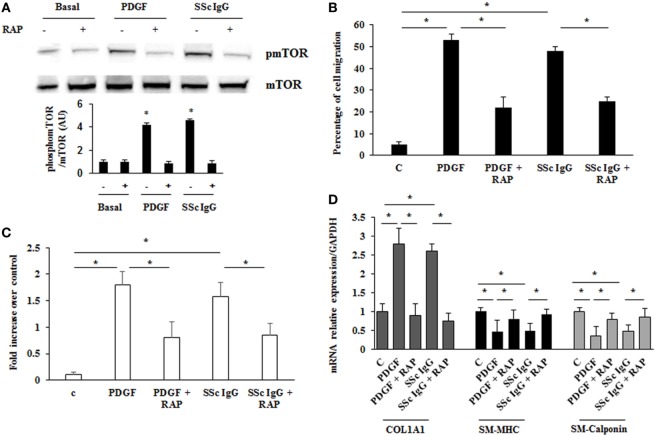
**Modulation of systemic sclerosis (SSc) IgG effects by rapamycin**. **(A)** HPASMC were stimulated with PDGF (15 ng/ml) or SSc IgG (200 µg/ml; *n* = 11) for 24 h in the presence or absence of rapamycin (10 nM) added 48 h before. Phosphorylation of mTOR was detected by immunoblotting, and anti-total mTOR antibody was used as a control for normalization. One representative experiment is shown in the upper panel. Densitometric analysis from three independent experiments is reported in the lower panel. Data are presented as mean ± SD. *p < 0.05. **(B)** Migration was analyzed through wound healing scratch test. Cells were scraped and stimulated with PDGF (15 ng/ml) or single SSc IgG (200 µg/ml; *n* = 11) in the presence or absence of rapamycin (10 nM) added 48 h before. After 24 h, the gap size was measured and expressed as a percentage of the original wound. Data are presented as mean ± SD of five independent experiments. *p < 0.05. **(C)** HPASMC cells treated with PDGF (15 ng/ml) or single SSc IgG (200 µg/ml; *n* = 11) for 48 h in the presence or the absence of rapamycin (10 nM) added 48 h before. Proliferation was determined by 5-bromo-2′-deoxyuridine assay and expressed as fold increase over controls. Data are presented as mean ± SD of five independent experiments. *p < 0.05. **(D)** HPASMC were treated with rapamycin and then exposed to PDGF (15 ng/ml) or SSc IgG (200 µg/ml; *n* = 11) for 24 h. COL1A1, smooth muscle-myosin heavy chain, and SM-calponin mRNA levels were measured by real-time PCR. Data are presented as mean ± SD of three independent experiments. *p < 0.05. The IgG from each subject was single tested.

Mammalian target of rapamycin (mTOR) is a serine/threonine kinase that acts as a major activator of cell growth and proliferation. mTOR has also been shown to play a role in proliferation of vascular smooth muscle cells and when dysregulated has been implicated in vascular remodeling (39). To evaluate the role of mTOR as a possible downstream effector in stimulated HPASMC, we treated the cells with PDGF (15 ng/ml) or distinct SSc IgG (200 µg/ml; *n* = 11) for 24 h in the presence or absence of rapamycin (10 nM), a specific inhibitor of mTOR added 48 h before. Phosphorylation levels of mTOR were higher in samples incubated with PDGF or SSc IgG compared to unstimulated cells and were reverted by rapamycin (Figure 7A). Moreover, the treatment with rapamycin significantly decreased the migration rate (Figure 7B), proliferation (Figure 7C); and COL1A1 expression (Figure 7D) and restored the levels of SM-MHC, and SM-calponin (Figure 7D) in HPASMC incubated with PDGF or SSc IgG. These results demonstrated that mTOR is a crucial downstream signaling of SSc IgG effects *via* PDGFR.

## Discussion

Vascular smooth muscle cells are highly specialized cells whose principal functions are contraction and regulation of blood vessel tone. In the mature arterial wall, they exhibit a contractile phenotype characterized by low rate of proliferation and expression of specific markers such as α-SMA, SM-MHC, and SM-calponin. In response to vascular injury, they can dedifferentiate into a proliferative and synthetic phenotype. This phenotypic switching contributes to intimal hyperplasia and has been linked to the development and progression of several vascular disease (38). The functional state of smooth muscle cells is controlled by a complex combination of local environmental cues and epigenetic programs that influences synthetic or contractile, as well as intermediate phenotypes (40–44).

In this study, we analyzed the expression of distinct functional markers of smooth muscle cells from human pulmonary arteries exposed *in vitro* to anti-PDGFR autoantibodies from SSc patients. In our experimental conditions, the data show that HPASMC acquire a synthetic phenotype characterized by higher growth rate, migratory activity, type I collagen gene expression, and minimal expression of markers characteristic of the contractile phenotype such as SM-MHC and smooth muscle-calponin. Thus, our findings indicate that anti-PDGFR autoantibodies may contribute not only to the development of SSc fibrotic lesions (23, 26) but also to the development of the vascular features. However, it is important to point out that our data do not allow to establish whether the new phenotype is due to the conversion of normal contractile vascular smooth muscle cells to a less differentiate state or to the expansion of medial-derived multipotent vascular stem cells (45).

Furthermore, since none of the SSc IgGs were from patients with pulmonary arterial hypertension, it seems that the impact of anti-PDGFRα autoantibodies on vascular smooth muscle cells reflects a general phenomenon, that is, scleroderma vasculopathy, and the association of their serum levels to specific clinical features (pulmonary arterial hypertension, digital ulcers, and scleroderma renal crisis) must be addressed in a larger cohort of SSc patients.

The activation of PDGFR by SSc IgG was both selective and ROS dependent since the presence of AG 1296 or NAC reduced the proliferation and migration of HPASMC and increased the expression of the differentiation markers. Our data are at variance with the findings reported by Arts et al. (46) who demonstrated that in smooth muscle cells SSc IgG engaged PDGFR leading to the activation of the epidermal growth factor receptor through a PDGFR-independent pathway. Furthermore, even if SSc IgG induced a profibrotic change in smooth muscle cells, collagen gene expression and cell proliferation were not influenced. Finally, some crucial experimental variables such as the source of the cells (aortic rat versus human pulmonary smooth muscle cells) and the timing of stimulation may explain the difference between their and our data.

Reactive oxygen species are known to mediate a variety of intracellular process, and the identification of the sources of these ROS has facilitated our understanding of a number of biological functions. Here, we demonstrated that the SSc IgG-induced abnormal oxidative stress through NOX4 activation facilitated the synthetic phenotype of human pulmonary artery smooth muscle cells. Interestingly and similar to what occurs in SSc fibroblasts (36), SSc IgG increased the expression of NOX4, while NOX1 was unaffected. These findings are the exact opposite of what reported by Lassègue et al. (47). If this discrepancy is to be ascribed to the different cell line and methodology used or indicates a different pathophysiological role of smooth muscle cells, NOX isoforms in response to distinct environmental cues remain to be established. Furthermore, at variance with what we found in normal fibroblasts (36), in HPSMC, SSc IgG did not induce NOX2, suggesting that individual NOX enzymes may play distinct roles between cell types. Thus, the precise interplay of NOX2 and NOX4 in HPSMC following SSc IgG stimulation needs further studies.

Cell proliferation and migration are thought to be a critical step in fibrogenesis, and since there is a strict interplay between mTOR and redox-based signaling (48, 49), we addressed the contribution of mTOR signaling to these phenotypes in cells exposed to ROS inducing stimuli.

mTOR is a serine/threonine kinase that consists of two distinct complexes mTORC1 and mTORC2. Growth factors, nutrients, DNA damage, and hypoxia among others regulate mTOR, which, once activated, phosphorylates multiple downstream signals playing a key role in cellular protein synthesis, control of eukaryotic cell growth, and proliferation, including cell cycle progression, differentiation, protein degradation, apoptosis, and angiogenesis (50). The role of mTOR in the vasculature is subject to intense investigation, particularly in the field of pulmonary arterial hypertension, where evidence has been provided of its importance in the events leading to proliferation and survival of pulmonary vascular cells and its modulation by growth factors, vasoactive agents, vascular Ca^2+^ channels, and chronic hypoxia (50).

Interestingly, Eid et al. (51), in a different experimental system, have shown that the pharmacological inhibition of mTOR with rapamycin decreases NOX4 activity, ROS production, and podocytes apoptosis induced by high glucose, suggesting that rapamycin may represent a therapeutic modality of diabetic kidney disease (51). In line with their observations, we show that ROS-inducing SSc IgG activated mTOR, and its pharmacological inhibition with the antifungal macrolide rapamycin blocked PDGF and SSc IgG ability to promote crucial functions of vascular smooth muscle cells such as proliferation, migration, and collagen production, implying that mTORC1 mediated the phenotypic conversion.

These findings underscore the potential importance of mTOR in the pathogenesis of SSc, also in light of studies conducted on fibroblasts *in vitro* and in experimental models of fibrosis. mTOR activation contributes to type I collagen production by dermal fibroblasts (52, 53), and rapamycin improves skin fibrosis in two mouse models of fibrosis (54). Treatment of a small cohort of SSc patients with rapamycin was safe, but the efficacy was not impressive (55). However, the clinical benefit of a more efficient mTOR blockade should be explored exploiting the novel information regarding mTOR signaling targets.

In conclusion, our study demonstrates that stimulatory autoantibodies targeting PDGFR activate smooth muscle cells and may, thus, contribute to the development of SSc vascular lesions.

However, to better define their role in the pathogenesis of the individual SSc vascular manifestations, such as pulmonary arterial hypertension, digital ulcers, and SSc renal disease, it is necessary to study vascular smooth muscle cells of distinct organs, those isolated from tissues of SSc patients, and to establish whether there is indeed an association between the serum levels of anti-PDGFRα autoantibodies and particular vascular features. Nevertheless, the present findings and those already published (23, 24, 27) highlight the importance of an early treatment of scleroderma aimed at downregulating B cell immune response.

## Author Contributions

SS designed the experimental work analyzed results and wrote the first draft of the manuscript. DA, TS, and MR performed experimental work and analyzed results. CF and DF selected and provided samples and analyzed clinical data. GM, CP, CT, and AGR provided the monoclonal autoantibodies. AF contributed to write the final revised version of the manuscript. AGA designed, supervised, evaluated the experiments, and wrote the final version of the manuscript. All the authors read and approved the manuscript.

## Conflict of Interest Statement

The authors declare that the research was conducted in the absence of any commercial or financial relationships that could be construed as a potential conflict of interest.
