# Corrigendum: Agonistic Anti-PDGF Receptor Autoantibodies from Patients with Systemic Sclerosis Impact Human Pulmonary Artery Smooth Muscle Cells Function *In Vitro*

**DOI:** 10.3389/fimmu.2017.00381

**Published:** 2017-04-12

**Authors:** Silvia Svegliati, Donatella Amico, Tatiana Spadoni, Colomba Fischetti, Doreen Finke, Gianluca Moroncini, Chiara Paolini, Cecilia Tonnini, Antonella Grieco, Marina Rovinelli, Ada Funaro, Armando Gabrielli

**Affiliations:** ^1^Clinica Medica, Dipartimento di Scienze Cliniche e Molecolari, Università Politecnica delle Marche, Ancona, Italy; ^2^Dipartimento di Scienze Mediche, Università di Torino, Torino, Italy

**Keywords:** systemic sclerosis, autoantibodies, vascular smooth muscle cells, platelet-derived growth factor, synthetic phenotype

In the original article, we neglected to add Ada Funaro as an author. The correct author list and affiliation should be

Silvia Svegliati^1†^, Donatella Amico^1†^, Tatiana Spadoni^1†^, Colomba Fischetti^1^, Doreen Finke^1^, Gianluca Moroncini^1^, Chiara Paolini^1^, Cecilia Tonnini^1^, Antonella Grieco^1^, Marina Rovinelli^1^, Ada Funaro^2^ and Armando Gabrielli^1^*

^1^Clinica Medica, Dipartimento di Scienze Cliniche e Molecolari, Università Politecnica delle Marche, Ancona, Italy

^2^Dipartimento di Scienze Mediche, Università di Torino, Torino, Italy

## Author Contributions

SS designed the experimental work analyzed results and wrote the first draft of the manuscript. DA, TS, and MR performed experimental work and analyzed results. CF and DF selected and provided samples and analyzed clinical data. GM, CP, CT, and AGR provided the monoclonal autoantibodies. AF contributed to write the final revised version of the manuscript. AGA designed, supervised, evaluated the experiments, and wrote the final version of the manuscript. All the authors read and approved the manuscript.

The authors apologize for this oversight.

This error does not change the scientific conclusions of the article in any way.

The original article has been updated.

## Conflict of Interest Statement

The authors declare that the research was conducted in the absence of any commercial or financial relationships that could be construed as a potential conflict of interest.

